# Neonatal mortality in a public referral hospital in southern Haiti: a retrospective cohort study

**DOI:** 10.1186/s12887-022-03141-4

**Published:** 2022-02-07

**Authors:** Alka Dev, Michelucia Casseus, Wilhermine Jean Baptiste, Emma LeWinter, Patrice Joseph, Peter Wright

**Affiliations:** 1 The Dartmouth Institute for Health Policy and Clinical Practice, Dartmouth College, Lebanon, NH USA; 2 Dartmouth-Hitchcock Medical Center, Lebanon, NH USA; 3 Hopital Immaculae Conception, Les Cayes, Haiti; 4 GHESKIO Centers, Port au Prince, Haiti

**Keywords:** Neonatal mortality, Prematurity, Hypoxia, Sepsis, Haiti, Newborn care, Neonatal morbidity, Hospital mortality

## Abstract

**Background:**

Haiti has the highest rate of neonatal mortality in the Latin America and Caribbean region. While the rate of facility births in Haiti has doubled over the past two decades, there have been no comparable reductions in maternal or neonatal mortality. Little data is available on the clinical characteristics of complications and morbidities among newborns requiring hospitalization after birth and their contribution to neonatal mortality. There is a need to better understand the status of newborn clinical care capacity in Haiti to prioritize training and resources.

**Methods:**

We performed a retrospective observational cohort study of neonates admitted to a large public referral hospital in southern Haiti in the first 2 years of operation of a new neonatal unit that we established. All neonate cases hospitalized in the unit in these 2 years were reviewed and analyzed to identify their clinical characteristics and outcomes. Multivariable logistic regression was used to identify independent risk factors of hospital mortality. We present the outcomes for 1399 neonates admitted to the unit during August 2017 and August 2019.

**Results:**

The leading cause of death was prematurity, followed by hypoxia and infection. Inborn neonates had better rates of hospital survival than those born elsewhere; they were also more likely to be born via cesarean section and to be admitted immediately following birth. There were no differences between the proportion of premature or low-birth-weight babies born at the hospital or elsewhere. Mortality in the second year of the unit’s operation was 12%, almost half that of the first year (21%). Multivariable regression analysis showed that mortality was consistently higher among premature and very low birthweight babies.

**Conclusions:**

With modest investments, we were able to halve the mortality on a neonatal unit in Haiti. Resources are needed to address prematurity as an important outcome since hospital mortality was significant in this group. To this end, investment in uninterrupted supplies of oxygen and antibiotics, as well as ensuring adequate newborn resuscitation, infection control, laboratory testing, and timely morbidity and mortality reviews would go a long way toward lowering hospital mortality in Haiti.

## Background

Haiti has struggled with multiple development setbacks over the past few decades, impeding its capacity to respond effectively to the healthcare needs of its people [1]. The country’s neonatal mortality rate, estimated to be 25 per 1000 live births, is the highest in the Latin America and Caribbean region compared to the regional rate of 9 per 1000 births [2]. Of the 16,427 Haitian infants who died before their first birthday in 2019, 60% died within the first 28 days of life, the neonatal period; 70% of these neonatal deaths occurred in the first week [3]. With an increase in facility births in low-income countries, survival for neonates is generally expected to improve. In a systematic review of the effects of health-facility delivery on neonatal mortality, Tura and colleagues showed that neonatal mortality was 29% lower in facility births than home births [4]. While the rate of facility births in Haiti has doubled to 40% over the past two decades, this has not led to comparable reductions in maternal or neonatal mortality and hospital neonatal mortality in Haiti remains high [5–7]. Further, women report disrespect, maltreatment, and isolation at health facilities, impacting their decision to seek medical care and preference for delivering at home [8, 9]. The national health budget declined four-fold between 2004 and 2016, with greater reliance on international assistance and out-of-pocket payments even though poverty is a severe barrier to accessing medical care [10]. Thus, maintenance of even the most basic public-health functions has been challenging given the current financing and governance structures. Understandably, Haiti lags far behind other countries in achieving its public health targets.

Haiti has a three-tiered health care system designed to improve primary care access at the community level [11–13]. Maternal and newborn health services are overseen by the Department of Family Health within the Ministry of Public Health and Population to ensure that every birth is attended by a qualified provider. Tier 1, primary care, has 3 levels. The first level includes dispensaries that provide essential medicines and are staffed by community health workers – screening for risk factors during pregnancy and newborn vaccinations are available here. Level 2 includes Health Centers (with or without beds) that are staffed by medical personnel who provide basic primary care services, screening for sickle cell anemia, management of risk factors, HIV testing and treatment, low- to moderate-risk vaginal deliveries, and follow-up of sick newborns are available here. Level 3 includes Community Reference Hospitals offering basic inpatient clinical services – high-risk vaginal deliveries are done here. Tier 2, secondary care, includes Departmental Hospitals which serve as the reference institution for each of the 10 Departments – they can provide cesarean sections and management of newborn referral cases from the lower levels. Tier 3, tertiary care, includes academic hospitals and specialized national institutions – their role in maternal and newborn care is to provide specialty services but Haitian women also seek care here for normal deliveries. While 91% of Haitians live within 5 km of a primary care facility, most of these facilities are cited for inefficiency and low-quality care due to low productivity, absenteeism, and stockouts [14]. Vast disparities remain between urban and rural facilities in terms of obstetric service delivery readiness and newborn care [15, 16].

The lack of appropriate prenatal care, the low quality of obstetric care, and high rates of eclampsia, obstructed labor, untreated infection, and preterm delivery are persistent factors behind the high rates of maternal and neonatal mortality in Haiti [6, 7, 17, 18]. In a recent analysis, we reported that 2–5% of deliveries in hospitals in southern Haiti suffered from eclampsia, which was likely an underestimation due to poor diagnostic capacity and data quality [19]; an eclampsia rate of 23% was reported in another Haitian hospital with better electronic record-keeping [20]. Lack of medical supervision, essential newborn care services, and an ability to respond to emergent situations during birth can contribute to birth asphyxia, infection, and prematurity-related complications, which are major causes of neonatal mortality in low-income countries [21]. Rural hospitals in Haiti often lack the capacity to care for newborns after birth due to gaps in essential infrastructure, equipment, supplies, medicines, and staff trained to implement up-to-date protocols and guidelines [16]. In addition to improving skilled care at birth, we need to generate evidence regarding the provision of skilled care to neonates requiring hospitalization. Case management in neonatal care units can inform service delivery and quality improvement initiatives for newborn morbidity, mortality, and survival [22].

To care for newborns requiring hospitalization in Haiti, we worked with the leadership at Hopital Immaculae Conception (HIC), a departmental hospital in *Sud*, to establish a neonatal care unit for newborns needing medical care after birth. The objective of this study was to conduct a retrospective review of neonatal admissions during the first 2 years of the unit’s operation to identify risk factors for hospital neonatal mortality, determine if survival improved over time, and inform newborn care based on our analysis.

## Methods

### Study setting

**Fig. 1:**
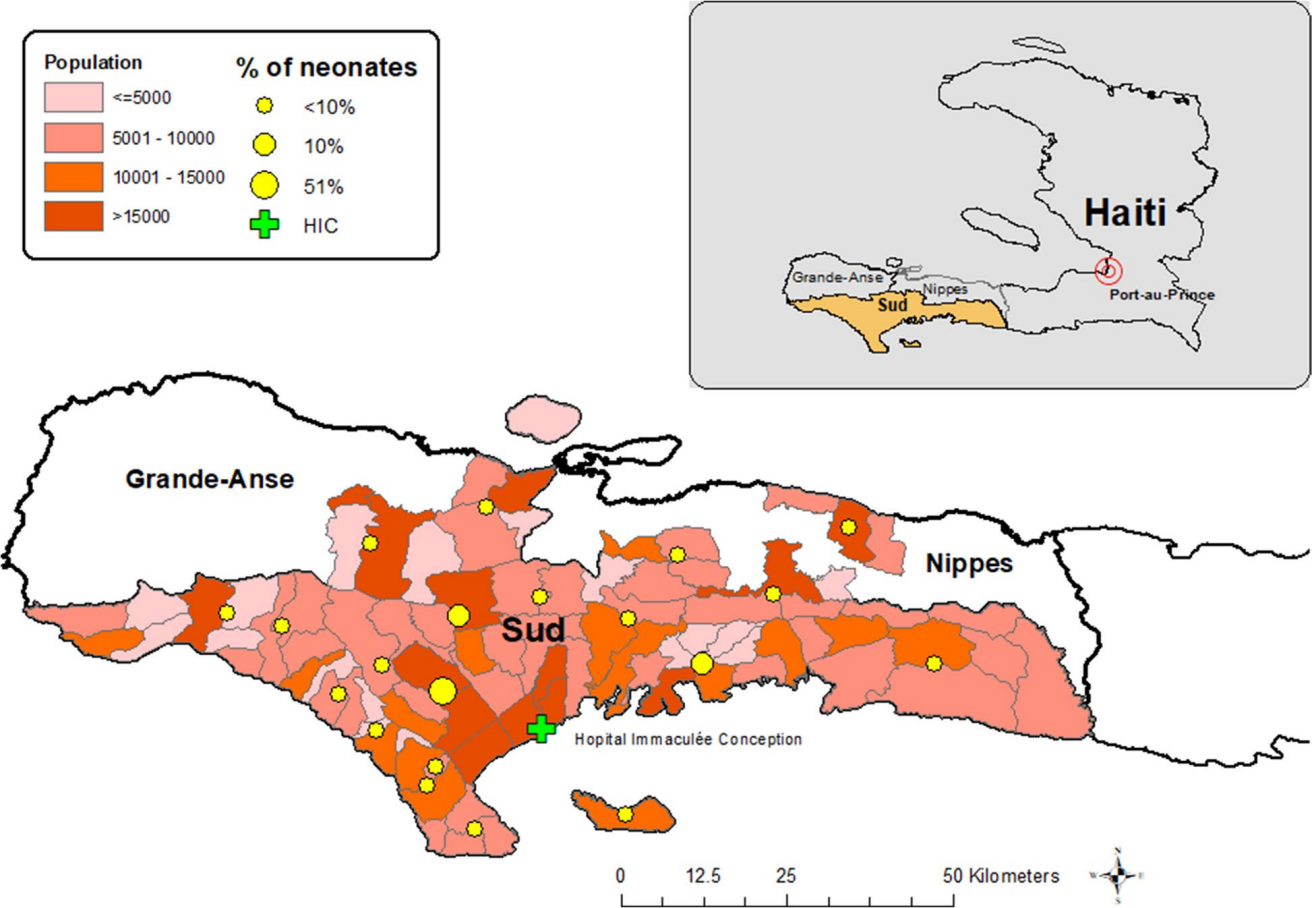
Location of Hopital Immaculae Conception, its sending communes, and population density in surrounding areas [19]. The map shows the population density (by sub-commune) and the percentage of neonates coming from each of the communes in the region. The highest proportion (51%) of neonates were admitted from the area in the immediate vicinity of HIC. The remaining neonates were spread out across the Sud region, some coming from neighboring departments of Nippes and Grand Anse

HIC is the main departmental referral hospital (secondary care), located in *Les Cayes* and serving a population of 774,976 (Fig. 1). There are 18 communes (or sub-departmental administrative units) in *Sud*, of which *Les Cayes* is the largest with an approximate population of 140,327 people [23]. The maternity ward has an annual volume of 3000–4000 deliveries [19]. At the time of our project, the maternity ward had two beds in the delivery room and was staffed by a midwife or a nurse who attended deliveries and provided newborn resuscitation, as needed. Obstetricians attended some of the more complicated deliveries and performed cesarian sections. A warmer was available but never used as the staff was under the assumption that it did not work because a message showing it was warming up would appear in red and staff assumed it was an error message and turned it off. We corrected this misunderstanding but did not document if the warmer was used consistently afterward. Normal procedures following a low-risk birth included weighing the baby, giving vitamin K, and applying ophthalmic tetracycline ointment. There was no oxygen in the delivery room, but suction and/or resuscitation with a bag valve mask was performed as needed. If a baby needed oxygen, an oxygen concentrator was used if there was electricity. No intubation was ever performed on the delivery ward. Babies in need of oxygen or intubation were referred out and likely did not survive the journey. Water and soap were available, but hand sanitizer use was more common.

Most women arrived at the maternity ward shortly before birth and after laboring at home. Accurate estimates of gestational age were generally not available. Clinical assessment and the date of the last menstrual period were used to establish the gestational age to determine prematurity at the time of delivery. Approximately 15% of births were through cesarean section [19]. Breastfeeding was generally not initiated immediately after birth or promoted in the maternity ward. There was no specific staff assigned for newborn care nor adequate space, equipment, or material for case management. The pediatric unit was located next to the maternity ward and received newborns, but those in need of critical care were referred to a hospital that was more than 2 h away.

### Neonatal unit structure and function

In June 2017, a team from Dartmouth and GHESKIO, with funding from the Children’s Prize and the WK Kellogg Foundation, established the first neonatal care unit at HIC. The unit functioned at a Level IIA neonatal capacity, caring primarily for newborns who were ill with problems that were expected to resolve quickly with moderate risk of serious complications and no assisted ventilation [24]. Two pediatricians were appointed to provide care on an 8-bed unit, which was later expanded to 13 beds due to high demand. The hospital administration hired 8 nurses to staff the unit. Other support staff included a project coordinator, a data manager, a lab coordinator, three community health workers, and four cleaners. All staff was supported through project funding. Pediatricians alternated each week on inpatient and outpatient follow-up services. One pediatrician was on call every night and weekend. A third pediatrician provided vacation coverage as needed.

Renovation and expansion of the space, as well as provision of essential equipment, medications, oxygen, and supplies, were completed sequentially over 2 years. We note that there were two incubators in use but due to inconsistent power supply, these were not always reliable. Additional equipment purchased and provided through our grant included eight warmer-bassinets, three oxygen concentrators, four incubators, and a portable ultrasound (for maternity ward). Toward the end of our project, electricity was regularly supplied on the neonatal ward due to the installation of a new generator at the hospital. There were no ventilators on the ward. Although nursing and administrative staff were later integrated into the hospital’s operating budget, the initial investment and technical oversight were provided by our team. All staff reported to supervisors within the hospital and were integrated into the hospital’s human resource structure.

### Staff training and services

The two pediatricians (MC and WJB) received 3 months of supervised neonatal training at Dartmouth-Hitchcock Medical Center in the U.S., and GHESKIO and St. Boniface Hospital in Haiti. This included a master training in *Helping Babies Breathe* (HBB) at Dartmouth [25]. There was no postgraduate training available for neonatal care at HIC and no pediatric residents rotated through the hospital as it was not an academic hospital. Neonatal training for nurses was delivered at hiring and after 18 months of service by visiting neonatal nurses from the U.S. and Haiti. In the interim, a visiting Dartmouth pediatric resident provided on-the-job training to nurses in HBB and *Essential Care for Every Baby* [26] curricula, jaundice care, shift handoff, head-to-toe exams, intake assessments, and Kangaroo Mother Care (KMC). We also reviewed nursing charts and redesigned nursing forms to ease shift handoff, sign off on doctors’ orders, reduce redundant note-writing, and properly document medications. Nurses encouraged all mothers and fathers to provide KMC. Breastfeeding was encouraged and promoted when a neonate was admitted and if the mother was able to initiate. Manual breast pumps were provided to mothers in recovery after a cesarean or for neonates who were unable to latch on. The pediatricians and nurses made every effort to promote breastfeeding, but the lack of comfortable seating remained a barrier.

### Data sources and outcomes

Patients were admitted to the neonatal care unit from the maternity ward at HIC or were born at other facilities/homes in the region and transferred to the unit by their families. In a small number of cases, newborns were transferred by ambulance from referring facilities. The admitting staff included both pediatricians and nurses. A daily electronic register documenting the date of birth, date of admission, place of birth, weight at admission, prematurity status, major health issue(s), length of stay, and outcome for all infants admitted to the unit, was maintained by the pediatricians in MS Excel©. Data were reviewed quarterly by the pediatricians and Dartmouth-GHESKIO teams to track progress, review mortality cases, and identify major bottlenecks. The final database for this study was a compilation of all admissions to the unit between August 2017 and August 2019. The outcome of interest was an inpatient neonatal death.

### Diagnostic criteria

Based on the assessment of the pediatricians, primary diagnoses were established based on the International Classification of Diseases and Related Health Problems (ICD-10) maintained by the WHO [27]. Due to limited laboratory and diagnostic resources, the pediatricians were unable to confirm their diagnoses with more than minimal blood work. Microbiologic definitions, other than HIV and syphilis screening tests, were not available. Prematurity was defined as gestational age less than 37 weeks, which was established using the Ballard Score Maturational Assessment of Gestational Age in Newly Born Infants [28]. Low birth weight (LBW) at admission was defined as weight less than 2500 g and very low birth weight (VLBW) as less than 1500 g. A perinatal hypoxia diagnosis was given to babies that had “no cry at birth,” meconium aspiration, surfactant deficiency, fetal distress, and/or secondary hypoxic injury, such as Hypoxic Ischemic Encephalopathy (HIE). Acute respiratory distress syndrome was reserved for preterm newborns with severe illness, such as sepsis, pancreatitis, or pneumonia, in addition to hypoxia. HIE was diagnosed a few hours after birth by using Sarnat classification and the birth history context [29]. Jaundice was diagnosed based on clinical signs such as the color of the skin and mucosa, and grade by localization of the yellow coloration in the body or mucosa and level of blood Bilirubin. Treatment for higher-than-normal levels of Bilirubin was phototherapy and sometimes, phenobarbital. Hypoglycemia was diagnosed using a dipstick test of blood sugar at admission and clinical signs like lethargy and poor suction. Treatment was administered in the form of IV Dextrose 10: 2 cc/kg stat. Another test was administered after 15 min and if positive, another bolus of Dextrose 10 was administered.

### Exclusion criteria

**Fig. 2:**
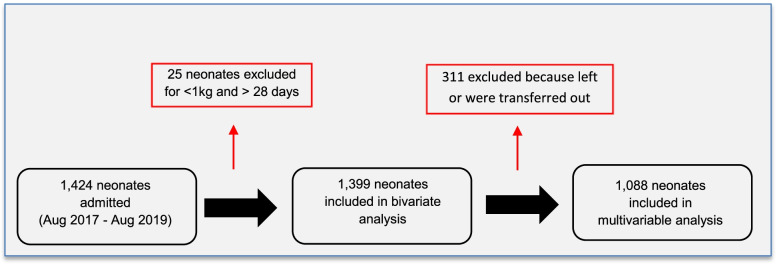
Sample calculation

A flow chart shows the derivation of the sample and reasons for exclusion (Fig. 2). Very small neonates weighing less than 1 kg (*n* = 12) were excluded from the dataset due to their exceptionally poor survival prognosis. Infants older than 28 days were also excluded (*n* = 11). All neonates taken home against medical advice or transferred out of the unit were excluded from the multivariable analysis, but we compared their baseline characteristics to identify any patterns among those who left. Parents’ reasons for leaving early were numerous: having other children to care for at home; timing with major festivals; lack/cost of room and board for families; and political unrest and insecurity. We suspected that some parents also wanted the child to receive traditional medicine at home or were not confident that the baby could recover at the hospital.

### Analyses

Neonates who were born at HIC were compared to those who were born elsewhere (at home or another facility) to identify any observable differences in neonatal characteristics using a Pearson’s chi-squared test for categorical variables and Student’s t-test for mean length of stay. (Table 1). Next, we tested for bivariate relationships between those who died and those who were discharged, excluding neonates whose parents took them home early against medical advice, to identify significant factors contributing to mortality in the neonatal unit (Table 2). Multivariable analysis was performed on the same dataset to calculate adjusted odds ratios of dying on the ward.

**Table 1:** Baseline characteristics of 1399 neonatal inpatients at Hopital Immaculae Conception, Les Cayes by place of birth (2017–2019)

	Outborn	Inborn	Total	*p*-value
*N* (%)	477 (34.1)	922 (65.9)	1399 (100.0)	
Sex				0.164
Male	288 (60.4)	521 (56.5)	809 (57.8)	
Female	189 (39.6)	401 (43.5)	590 (42.2)	
Age at admission				**0.000**
Day of birth	128 (26.8)	578 (62.7)	706 (50.5)	
1–6 days after birth	219 (45.9)	271 (29.4)	490 (35.0)	
7–28 days after birth	130 (27.3)	73 (7.9)	203 (14.5)	
Timing of birth				0.804
Term	386 (80.9)	741 (80.4)	1127 (80.6)	
Preterm	91 (19.1)	181 (19.6)	272 (19.4)	
Outcome				**0.000**
Died	69 (14.5)	102 (11.1)	171 (12.2)	
Discharged	274 (57.4)	643 (69.7)	917 (65.5)	
Left against Dr’s orders	123 (25.8)	168 (18.2)	291 (20.8)	
Transferred	11 (2.3)	9 (1.0)	20 (1.4)	
Multiple gestations				0.472
Singleton	435 (91.2)	851 (92.3)	1286 (91.9)	
Twin or more	42 (8.8)	71 (7.7)	113 (8.1)	
Birthweight categories				0.233
1.0–1.4 kg	35 (7.3)	52 (5.6)	87 (6.2)	
1.5–2.4 kg	145 (30.4)	258 (28.0)	403 (28.8)	
2.5+ kg	297 (62.3)	612 (66.4)	909 (65.0)	
Type of Delivery				**0.000**
Vaginal	436 (91.4)	690 (74.8)	1126 (80.5)	
Cesarean	41 (8.6)	232 (25.2)	273 (19.5)	
Age of mother				0.172
< 18 years	17 (3.6)	45 (4.9)	62 (4.4)	
18–35 years	368 (77.1)	737 (79.9)	1105 (79.0)	
> 35 years	76 (15.9)	112 (12.1)	188 (13.4)	
Unknown	16 (3.4)	28 (3.0)	44 (3.1)	
Mean number of living children	1.3	0.98	1.09	**0.0001**
Mean length of stay (days)	4.3	4.3	4.3	0.977

**Table 2:** Case fatality by clinical diagnosis^a^

Diagnosis	Number admitted	Case fatality (%)	Proportion of all deaths (%)
Prematurity	182	61 (33.5%)	35.7%
Hypoxia	215	26 (12.1%)	15.2%
Suspected Infection	396	23 (5.8%)	13.4%
Hypoxia + Infection	113	13 (11.5%)	7.6%
Encephalopathy	71	26 (36.6%)	15.2%
Other	111	22 (19.8%)	12.9%
Total	1088	171 (15.7%)	100%

^a^Does not include those who left without discharge

### Patient and public involvement

As this study was based on retrospective data review, patients were not involved in choosing the methods or agreeing to plans for dissemination of the study results to parents and their communities.

## Results

### Neonatal characteristics

Half of the patients admitted to the neonatal unit at HIC were born to mothers from *Les Cayes*, the commune in which HIC is located (Fig. 1). Another 10% of babies were born to moms in the nearby *Torbeck* commune which has a birthing center with ambulance availability. The remaining cases came from the rest of *Sud* and communes in departments sharing a border with *Sud*. We did not ascertain why some of the neonates came from mothers in other departments; possibly they stayed with family living near HIC.

Table 1 presents the characteristics of all neonates admitted to the unit between August 2017 and August 2019 by place of birth. In all, 1399 neonates who were 28 days of age or younger and weighed more than 1 kg at admission were admitted to the unit; 66% were “inborn,” meaning born at HIC (*n* = 922), and the rest were “outborn,” meaning born at home or another facility (*n* = 477). There were very few differences in the recorded baseline characteristics of patients by place of birth. The proportions of boy versus girl neonates were similar for those born at HIC and elsewhere. Preterm birth complicated one-fifth of all admissions regardless of the place of birth. Less than 1 in 10 babies were from multiple gestation pregnancies. Differences in the neonates’ weights on admission and mothers’ ages were also insignificant by place of birth. Close to one-third of neonates were low or very low birth weight, regardless of the place of birth. A majority of mothers (79%) were between 18 and 34 years of age and 62 (4.4%) were 18 or under.

The three factors that were significantly different by place of birth were: survival status, the mode of delivery, and age at admission. Mortality was higher among outborn babies (14.5% versus 11.1% among babies born at HIC) and outborn babies were also more likely to be taken home against medical advice (25.8% versus 18.2% among babies born at HIC). The mode of delivery was vaginal for most births, but more so for outborn babies, which included home births (91.4% among outborn versus 74.8% among inborn). The majority of outborn babies were brought in within 1–6 days of birth (45.9%), compared to those born at HIC where the majority were transferred on the day of birth (62.7%). However, most of the admissions were made in the first week of life for all neonates. A higher proportion of births at HIC were through cesarean section (25%) compared to those born elsewhere (9%). Both sets of neonates had similar lengths of hospital stay (mean = 4.3 days).

Triage at birth to identify babies at risk was not consistently followed on the maternity ward as 17.3% of babies born at HIC were only admitted to the neonatal unit a day after birth. These babies were either kept in the maternity ward with their mother, where they deteriorated, or sent home and brought back by family members because of illness. Although we do not show the data, home births were more likely to be admitted later than births from other facilities.

### Characteristics of babies taken home against doctor’s orders

To analyze survival until discharge, we excluded babies who were taken home early by their parents and only included those who either died during their stay or were discharged by the attending pediatrician. Chi-square comparisons of included and excluded babies showed that there were no significant differences between those taken home early by parents versus those staying until discharge or death. Regarding birth weight, babies who were taken home early matched proportionally to those who remained until discharge or death (X^2^_3_ = 0.45, *p* = 0.930). There were no differences in the diagnoses of prematurity (X^2^_1_ = 0.86, *p* = 0.353), infection (X^2^_1_ = 0.03, *p* = 0.865), hypoxia (X^2^_1_ = 1.53, *p* = 0.215), or encephalopathy (X^2^_1_ = 1.04, *p* = 0.307) among the two groups. However, Student’s t-test showed that babies taken home early by their parents went home a day earlier on average (mean = 3.4 days) compared to those who stayed (mean = 4.5 days, t = 4.03, *p* = 0.0001). Mothers who left early with their babies had a higher number of live children at home (mean = 1.3) compared to those who stayed (mean = 1.0, t = − 2.41, *p* = .0001).

### Clinical characteristics

**Fig. 3:**
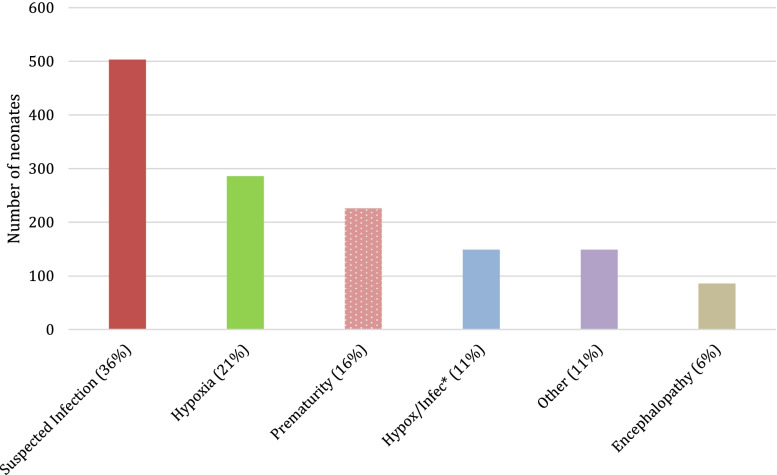
Major health issues experienced by neonates (*n*=1,399)

Diagnostic capacity was limited by the lack of confirmatory testing and information on maternal risk factors. The most common health issues experienced by the neonates included suspected infection, hypoxia, and prematurity; neonates often suffered from more than one complication (Fig. 3). Overall, 36% of neonates were suspected of having an infection, which was treated empirically with ampicillin and gentamycin and/or cefotaxime; 11% also shared a diagnosis of hypoxia in addition to the suspected infection. One in three neonates suffered hypoxia, with 11% also having complications from infection as noted above, and 6% with hypoxia and other complications (not shown separately). Nearly all premature infants were diagnosed as being hypoxic. Other health issues included: congenital malformations; obstetrical complications; jaundice; infections such as tetanus and meningitis; other pulmonary complications; seizures; cardiac conditions; and inappropriate feeding at home. Encephalopathy was clinically diagnosed in 6% of cases and included babies with Hypoxic Ischemic Encephalopathy and Hyperbilirubinemia Encephalopathy.

In all, 171 babies (15.7%) died during their stay at HIC (Table 2). In terms of case fatality, both prematurity and encephalopathy were quite fatal; in both cases, at least a third of infants with these diagnoses died. Another third of deaths occurred in hypoxic babies, suspected of having an infection or diagnosed with both hypoxia and infection. Other diagnoses included meningitis, congenital abnormalities, jaundice, tetanus, intestinal occlusion, and congenital syphilis.

### Hospital neonatal mortality vs. discharge

Table 3 presents results of chi-square tests of independence for hospital mortality versus discharge. As noted, preterm babies were much more likely to die than term babies (X^2^_1_ = 110.4, *p* = 0.000). There were notable differences by weight at admission and place of birth; VLBW and LBW babies were much more likely to die than to be discharged (X^2^_2_ = 126.0, *p* = 0.000) and babies born elsewhere and transferred to HIC also had higher mortality (X^2^_1_ = 7.3, *p* = 0.007) than inborn babies. Infants born via cesarean had better survival (X^2^_1_ = 11.1, *p* = 0.001). We also note that neonates admitted on the day of birth experienced higher mortality rates than those admitted a little later (X^2^_2_ = 28.4, *p* = 0.000). There was also an indication that babies born to teenage mothers under 18 years of age had higher mortality compared to other ages (X^2^_3_ = 13.7, *p* = 0.003). Finally, we compared mortality in the first year of the unit’s operation (August 2017 – August 2018) to the second year of the unit’s operation (September 2018 – August 2019) and found that mortality on the neonatal unit decreased by 40%, from 1 in 5 babies dying in the first year to 1 in 8 babies dying in the second year (X^2^_1_ = 16.1, *p* = 0.000). Other demographic characteristics, such as the neonate’s sex or being a twin were not correlated with mortality. Student’s-test showed that the difference in mean lengths of hospital stay was not significant by survival status but trending toward a somewhat longer stay for those who were discharged (t = 1.8, *p* = 0.075) and the number of live children was not a risk factor for hospital mortality.

**Table 3:** Risk factors for mortality among 1088 neonates at Hopital Immaculae Conception, Les Cayes (2017–2019)

	Discharged	Died	Total	*P*-value
*N* (%)	917 (84.3)	171 (15.7)	1088 (100.0)	
Timing of birth				**0.000**
Term	783 (90.1)	86 (9.9)	869 (100.0)	
Preterm	134 (61.2)	85 (38.8)	219 (100.0)	
Birthweight				**0.000**
1.0–1.4 kg	30 (43.5)	39 (56.5)	69 (100.0)	
1.5–2.4 kg	244 (77.2)	72 (22.8)	316 (100.0)	
2.5+ kg	643 (91.5)	60 (8.5)	703 (100.0)	
Sex				0.492
Male	521 (83.6)	102 (16.4)	623 (100.0)	
Female	396 (85.2)	69 (14.8)	465 (100.0)	
Place of birth				**0.007**
Outborn	274 (79.9)	69 (20.1)	343 (100.0)	
Inborn	643 (86.3)	102 (13.7)	745 (100.0)	
Multiple gestations				0.230
Singleton	841 (84.7)	152 (15.3)	993 (100.0)	
Twin or more	76 (80.0)	19 (20.0)	95 (100.0)	
Type of delivery				**0.000**
Vaginal	712 (82.4)	152 (17.6)	864 (100.0)	
Cesarean	205 (91.5)	19 (8.5)	224 (100.0)	
Age of mother				**0.014**
< 18 years	30 (68.2)	14 (31.8)	44 (100.0)	
18–35 years	741 (85.5)	126 (14.5)	867 (100.0)	
> 35 years	124 (84.9)	22 (15.1)	146 (100.0)	
Unknown	22 (71.0)	9 (29.0)	31 (100.0)	
Age of neonate at admission				**0.000**
Day of birth	435 (78.5)	119 (21.5)	554 (100.0)	
1–6 days after birth	336 (90.6)	35 (9.4)	371 (100.0)	
7–28 days after birth	146 (89.6)	17 (10.4)	163 (100.0)	
Mean length of stay (days)	4.6	3.9	4.6	0.075
Mean number of living children	1.00	1.12	1.08	0.343
Project timing				**0.000**
First year of unit operation	358 (79.0%)	95 (21.0%)	635 (100.0%)	
Second year of unit operation	559 (88.0%)	76 (12.0%)	453 (100.0%)	

Logistic regression models were supportive of our bivariate findings (Table 4). After adjusting for birth weight and other risk factors, preterm neonates had over twice the odds of dying compared to term babies (AOR = 2.38; *p* = 0.002). As expected, LBW and VLBW babies had higher odds of death when compared to normal-weight babies (NBW); LBW babies were 1.7 times as likely to die before discharge (*p* = 0.037) and VLBW babies were five times as likely to die (*p* = 0.000). The odds of dying were 2.2 times higher for babies referred to the hospital than those born at HIC (*p* = 0.000). Those born through cesarean section had a survival advantage (AOR = 0.47; *p* = 0.009). Mothers who were less than 18 years of age had higher odds of losing their neonate when compared to mothers who were 18–34 years old (AOR = 3.05, *p* = .004); the age of the mother was not a factor in mortality otherwise. Babies admitted 1–6 days after birth (AOR = 0.44; *p* = 0.001) or more than a week after birth (AOR = 0.40; *p* = 0.004) had less than half the odds of dying compared to babies admitted on the day of birth. Finally, after adjusting for observed risk factors, we established that the odds of a neonate dying in the second year of the unit’s operation was 56% lower than in the first half of the project (AOR = 0.44, *p* = 0.000).

**Table 4:** Odds of death in the hospital compared to discharge by risk categories

Died	Adjusted Odds Ratio (AOR)	(95% confidence interval)	*P*-value
Timing of delivery			
Term	*reference*		
Preterm	2.38	(1.39–4.06)	0.00**0**
Birthweight			
VLBW 1.0–1.4 kg	5.50	(2.58–11.76)	0.00**0**
LBW 1.5–2.4 kg	1.68	(1.03–2.75)	0.037
NBW 2.5 kg +	*reference*		
Place of birth			
HIC (inborn)	*reference*		
Other (outborn)	2.20	(1.43–3.37)	**0.000**
Type of delivery			
Vaginal	*reference*		
Cesarean	0.47	(0.27–0.82)	**0.009**
Age of mother			
< 18 years	3.05	(1.35–5.86)	**0.004**
18–34 years	*reference*		
> 35 years	1.31	(0.74–2.13)	0.327
Unknown	2.04	(1.08–6.50)	0.129
Age at admission			
Day of birth	*reference*		
1–6 days	0.44	(0.28–0.71)	**0.001**
7 days or more	0.40	(0.22–0.75)	**0.004**
Project timing			
First year of project	*reference*		
Second year of project	0.44	(0.30,0.64)	**0.000**

## Discussion

This study assessed the neonatal outcomes at a newly formed neonatal care unit in southern Haiti and showed overall mortality of 12.2% during the first 2 years of the unit’s operation. This mortality rate is midrange compared to mortality rates that have been reported in similar settings elsewhere, including southern Eritrea (6.5%), western Ethiopia (8.8%), north-central Nigeria (13.4%), western Haiti (14.5%), south-eastern Nigeria (19.4%), eastern Ethiopia (20.0%), Ghana (20.2%), and southern Ethiopia (25.0%) [6, 30–36]. If we exclude those who left the hospital early or were transferred out, we calculate a mortality rate of 15.7% among those with a known hospital outcome. We also had the opportunity to improve neonatal care through clinician training, renovation, provision of essential equipment and medications, and support for operational systems. There were significant improvements in survival to discharge over the 2 years, increasing from 79.0% in the first year to 88.0% in the second.

As seen in other low resource settings, mortality was highest among premature neonates; nearly 40% of all preterm babies died compared to 10% of term babies. Among premature neonates in Burundi, a similar disparity in inpatient mortality rate was observed; among those less than 32-weeks gestational age, 31% died compared to 11% among those who were 32–36 weeks’ gestation (11%) [37]. In India, over half of all neonatal deaths recorded in the National Neonatal Perinatal Database were among preterm babies [38]. Other published estimates of premature mortality are alarming as well; close to 50% of neonates born at 34 weeks of gestation and 70% of neonates born at < 32 weeks of gestation die in low-income countries [39, 40]. Specific gestational age was not available in our dataset. Other causes of death were similar as in other settings – with hypoxia and suspected infection leading the next third of all deaths, half of them attributable to infection. Comparatively, at St. Damien Hospital in Haiti, 23% of all neonates with sepsis died, although the odds of dying were 2.4 times higher for those who were treated empirically rather than culture-confirmed [7]. Sepsis-related neonatal mortality rates of 14.6–36.0% have been reported in other settings [41]. Thus, our sepsis results are comparable to those in other studies, although none of the cases were culture-confirmed. Our hypoxia-related death rates were somewhat lower than other settings – 15.2% for hypoxia and another 7.6% for hypoxia complicated by sepsis. Similar rates have been reported for asphyxia-related hospital mortality in Nigeria (24.1%) and Benin (20.0%) [33, 42]. Community-level rates of asphyxia-related deaths may be higher – as reported in Nepal (30.0%) [43].

Survival was better among neonates born at HIC, likely due to the higher risks associated with home births or births at facilities without cesarean section or other emergency obstetric services. Lower-level facilities in Haiti are poorly equipped to provide obstetric care, even if they manage labor and delivery [44]. We also found that the risk of dying was highest among babies admitted immediately after birth from HIC although we did not have sufficient data to determine why this may be the case. It is possible these neonates barely survived birth. Generally, mortality rates in favor of inborn babies have been well documented [45]. While the standard practice is to separate community transfers or outborn babies from inborn babies due to the risk of infection, we could not to do so which may have increased the risk of transmission from outborn to inborn neonates [46]. The proportion of preterm babies and those born with LBW or VLBW were similar for inborn and outborn infants; similar comparisons have been made in other settings [47].

Throughout the first 2 years, there was considerable variability but overall improvements in the delivery of neonatal care. This was driven in large part by the availability of antibiotics, laboratory testing, oxygen, and electricity; however, political instability, holidays, nursing schedules, and seasonal conditions also contributed to the unpredictability of unit conditions. Hot summer temperatures in a crowded unit were dangerous for infection control and hurricane-season rains often flooded the unit. Power outages were common at night and parents often slept on the floor of the unit by the neonatal beds. During renovations, the beds were moved to different parts of the pediatric ward, which affected the time it took to walk the babies over from the maternity ward. Over time, we were able to improve many of these conditions by renovating the neonatal space, providing better-quality furniture and equipment, as well as air conditioning and a steady supply of antibiotics and oxygen. However, the situation remained precarious due to the lack of adequate financial support from the health authorities. Therefore, it is notable that despite these challenges, survival improved over the first 2 years.

Another unexpected challenge was poor communication between the maternity and neonatal units. There was no formal referral system, where critical maternal information about labor and delivery would be communicated to the neonatal team. Additionally, neonatal care providers can have a broad influence in a hospital setting by ensuring that midwives and delivery nurses are properly trained in neonatal resuscitation and that parents are informed about maintaining hygiene, initiating breastfeeding, and recognizing newborn illnesses. This was sporadically done at HIC and limited by the poor communication across the two wards. We believe this was reflective of insufficient staffing across the hospital which led to a culture of siloed care within departments. Communication between doctors and nurses was also stifled and the power to ask questions or ask for materials resources was tied to seniority and tenure. Infrastructure was poor across the hospital due to a piecemeal project-by-project approach to building capacity rather than a strategic plan to improve hospital conditions. Better funded programs, such as HIV/AIDS prevention and treatment, were better staffed, equipped, and more efficient.

### Strengths and weaknesses

There are some notable strengths to this work. Hospital neonatal mortality data are difficult to access in Haiti, so our study presents a unique opportunity to learn about mortality risks in this important population. We audited a whole patient database of hospital neonatal mortality and were able to document the outcomes over 2 years – also allowing for an assessment of changes in mortality over time. We benefitted from the tireless effort of the pediatricians to document each data point and to alert us of any discrepancies with due notice. Similarly, we were able to discuss data quality issues and address inconsistencies in real time. While we could not do anything about data that were missing altogether, we do trust that the recorded data were of high quality.

This study also had several limitations. Although improvements in infrastructure and service-delivery capacity have been associated with improved neonatal survival in other settings [22], we could not meaningfully analyze the impact of specific improvements in the unit’s infrastructure or health service delivery, including the provision of free medications, as these improvements were made against the backdrop of ongoing political instability. Similarly, we did not track changes in nurse staffing, electrical supply, and other service-delivery parameters set by the hospital that were beyond our control and that likely led to fluctuations in care. There was considerable unpredictability in the hospital’s supply chain, which ultimately led us to secure therapeutics through the private sector. With less control over the supply chain, the providers could not ensure prescribed treatment for each neonate. We were not able to capture maternal risk factors, including intrapartum complications. Since the study was conducted among the newborns delivered at a public hospital, it cannot be generalized for the newborns delivered at home or in private hospitals and lower-level health centers. In addition, this study did not assess complications beyond the primary diagnosis.

## Conclusions

The potential to improve the quality of care in dedicated inpatient neonatal units in low- and middle-income countries cannot be ignored. There are many missed opportunities to provide effective interventions to neonates in health facilities. First, triage at birth to identify newborn distress must be prioritized to ensure a timely response, especially when treatment is available at the facility. Improving communication between the maternity and neonatal units, with a pediatrician attending complicated deliveries to identify neonates who are at risk of distress and illness, can improve the potential for recovery with earlier intervention. Second, considering the higher mortality among preterm babies, training for preterm care, especially for nurses, must be undertaken. Essential equipment, supplies, and medications, such as warmers, antibiotics, and an uninterrupted oxygen supply, should be made available in the very least. Culture confirmation capacity for suspected infections would improve care tremendously. And finally, Haiti must invest significant financial resources in the operation of newborn units, staffed with skilled personnel, at any hospital with a high-volume maternity ward or one that that receives referrals for complicated deliveries. With modest interventions, we were able to improve neonatal outcomes which is encouraging as we attempt to lower neonatal mortality in Haiti and beyond.

## Data Availability

The datasets used and/or analyzed during the current study are available from the corresponding author on reasonable request.

## Abbreviations

HIC: Hopital Immaculae Conception
ICD: International Classification of Diseases and Related Health Problems
LBW: Low Birthweight
NBW: Normal Birthweight
VLBW: Very-Low Birthweight
